# Non-cancer Causes of Death Following Initial Synchronous Bone Metastasis in Cancer Patients

**DOI:** 10.3389/fmed.2022.899544

**Published:** 2022-06-02

**Authors:** Yao Xu, Basel Abdelazeem, Kirellos Said Abbas, Yile Lin, Haixiao Wu, Fei Zhou, Karl Peltzer, Vladimir P. Chekhonin, Shu Li, Huiyang Li, Wenjuan Ma, Chao Zhang

**Affiliations:** ^1^Key Laboratory of Cancer Prevention and Therapy, National Clinical Research Center for Cancer, Tianjin's Clinical Research Center for Cancer, Tianjin Medical University Cancer Institute and Hospital, Tianjin, China; ^2^The Sino-Russian Joint Research Center for Bone Metastasis in Malignant Tumor, Tianjin, China; ^3^McLaren Health Care, Flint, MI, United States; ^4^Department of Internal Medicine, Michigan State University, East Lansing, MI, United States; ^5^Faculty of Medicine, Alexandria University, Alexandria, Egypt; ^6^Tianjin Medical University General Hospital, Tianjin, China; ^7^Department of Psychology, University of the Free State, Bloemfontein, South Africa; ^8^N. P. Serbsky National Medical Research Centre of Psychiatry and Narcology, Ministry of Health of the Russian Federation, Moscow, Russia; ^9^Department of Public Service Management, School of Management, Tianjin University of Traditional Chinese Medicine, Tianjin, China

**Keywords:** bone metastasis, non-cancer cause, cardiovascular, mortality, Surveillance Epidemiology and End Results (SEER) database

## Abstract

**Purpose:**

To investigate the non-cancer causes of death (COD) in cancer patients with synchronous bone metastasis (BM) that is based on the Surveillance, Epidemiology, and End Results (SEER) database.

**Methods:**

The retrospective cohort study included malignant cancer patients with synchronous BM diagnosed from 2010 to 2018 in the SEER database. The frequencies and proportion of non-cancer COD were calculated and analyzed in different genders, ages, and races subgroups.

**Results:**

A total of 97,997 patients were deceased and included into the current study and 6,782 patients were died of non-cancer causes with a male predominance (*N* = 4,515, 66.6%). Around half of deaths (*N* = 3,254, 48.0%) occurred within 6 months after diagnosis while 721 patients were deceased after 3 years. Lung and bronchus cancer, prostate cancer, breast cancer, kidney and renal pelvis cancer, and liver cancer were proved to be the top five cancer types resulting in non-cancer caused death. Cardiovascular and cerebrovascular diseases were the leading non-cancer cause of death (*N* = 2,618), followed by COPD and associated conditions (*N* = 553) and septicemia, infectious and parasitic diseases (*N* = 544). Sub-analyses stratified by gender, age and race were performed and the similar results with slightly difference were observed.

**Conclusions:**

Cardiovascular and cerebrovascular diseases were the main non-cancer cause of death in cancer patients with synchronous BM. Other non-cancer causes included COPD, septicemia, infectious and parasitic diseases, and so on. These findings should be considered by physicians. Physicians can counsel cancer patients with BM regarding survivorship with death causes screening and focus on prevention of non-cancer deaths.

## Introduction

Bone is a common metastatic site for many primary malignant tumors such as breast, lung, colorectal, and renal cancer (1, 2). The previous literature indicated that the prevalence of bone metastasis (BM) was second to liver and lung metastasis in cancer patients (2, 3). Although most BMs are asymptomatic and discovered incidentally, BM can present as bone pain, nerve compression, spinal cord, hypercalcemia, fracture, and impaired mobility (4). Moreover, BM diminished survival outcomes of cancer patients (5, 6). It was reported that more than 350,000 patients with BM die annually in the United States of America (USA) (2). A significant increase in mortality was observed in patients with BM. For example, there was a 6.5-fold increased risk of death in breast cancer patients with BM compared to those without BM (7). Based on the large population, a systematic study on the causes of death (COD) in cancer patients with BM can help improve the survival of cancer patients.

With the improvement of cancer-specific survival, the non-cancer COD raised more concerns recently. A study that included 1,105,607 identified deaths from 2000 to 2016 and concluded that the proportion of non-cancer caused deaths was increased over time in Korea (8). Heart diseases were the leading non-cancer cause, followed by cerebrovascular diseases and intentional self-harm in men, cerebrovascular diseases and diabetes mellitus in women (8). Compared with the Korean general population, the standardized mortality ratio (SMR) of suicide was up to 1.68 and 1.42 for male and female patients, respectively (8). In a retrospective study based on the Surveillance, Epidemiology, and End Results (SEER) database, a total of 26,168 metastatic prostate cancer patients were included and the COD were calculated (9). The study illustrated that the percentage of non-cancer causes was 16.7%. Cardiovascular diseases, chronic obstructive pulmonary disease, and cerebrovascular diseases were the top three non-cancer COD (9). Generally, the non-cancer causes varied across different patients and different types of primary tumor. Except for non-cancer COD in prostate cancer, other non-cancer causes were identified in multiple types of malignant tumors such as thyroid carcinoma and head and neck cancer (10, 11). To our best knowledge, there was still no comprehensive pan-cancer study to elucidate non-cancer COD in patients with BM.

In our study, we analyzed 126,817 patients from the SEER database to investigate the non-cancer COD after initial synchronous BM. We provided an overview of the non-cancer COD in different genders, ages, and races. Thus, our study may assist the physicians in redistributing cancer care toward the COD in patients with BM.

## Materials and Methods

### Data Sources and Study Population

We used the National Cancer Institute Surveillance, Epidemiology, and End Results (SEER) database to extract the data. The SEER database covers almost 28% of the US population. The current database name is *Incidence – SEER Research Data, 18 Registries (excl AK), Nov 2020 Sub (2000–2018) for SMRs*, which was released in April 2021. National Cancer Institute SEER^*^Stat software (www.seer.cancer.gov/seerstat) version 8.3.9 was used to generate original data. The patient information and the SEER^*^ Stat software were acquired from the SEER website (https://seer.cancer.gov/) after submitting the data agreement to the SEER administration. So informed concent from each patient was non-applicable.

**Figure 1:**
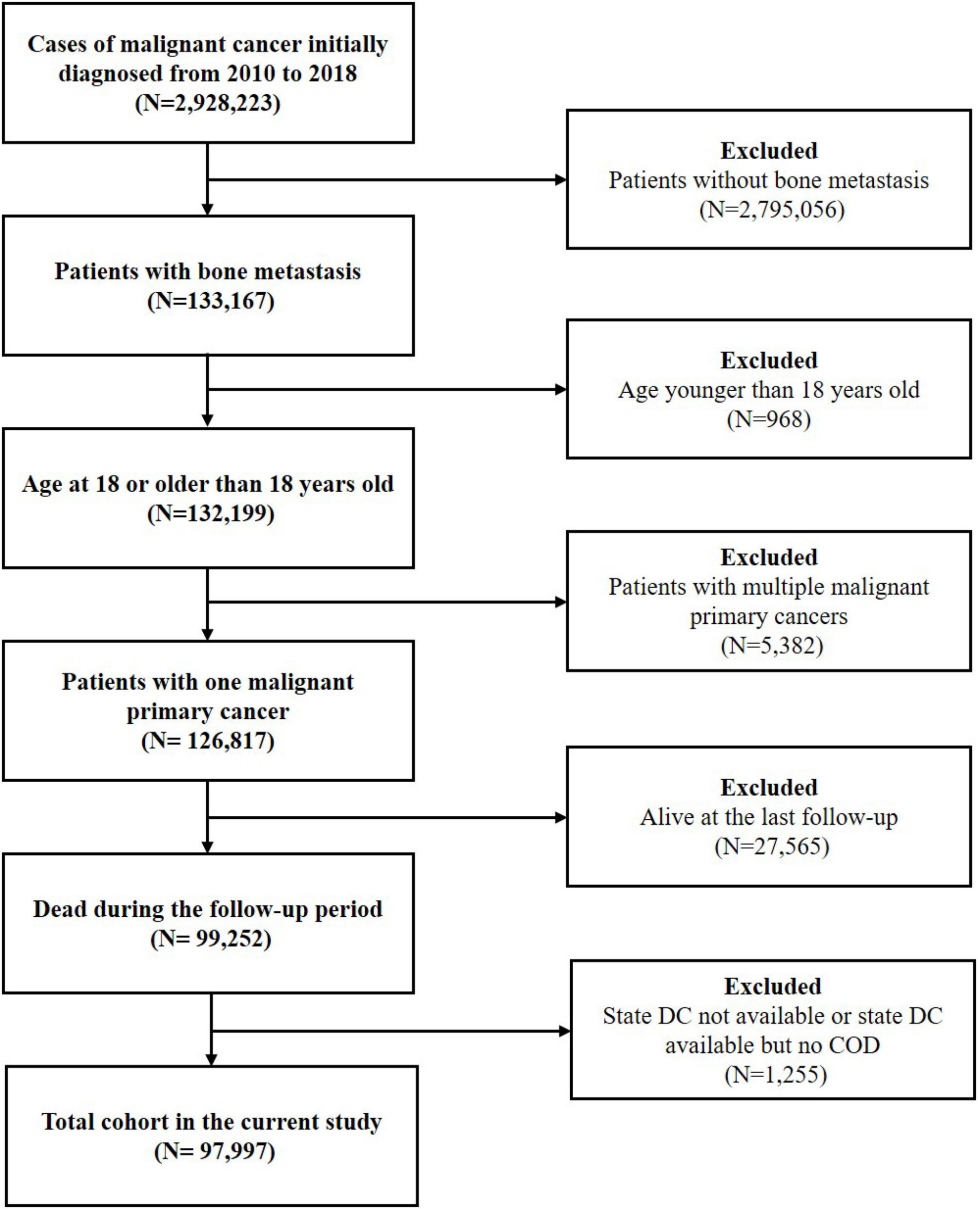
The flowchart of the present study.

The latency exclusion period was set as 1 month and patients with death certificates only and autopsy only were excluded. Malignant cancer patients with BM diagnosed from 2010 to 2018 were initially included. Since patients with metachronous BM were not recorded in the SEER database, only patients with synchronous BM were included. Then, patients younger than eighteen were excluded. To minimize the risk of bias, patients with multiple malignant primary cancers (≥2 cancers) were excluded. We selected cases with one malignant primary cancer according to the variable of “Multiple Primary Fields. Sequence Number.” Patients who were alive at the last follow-up and those without the records on the COD were excluded. Due to the restriction of cancer history, BM was regarded as being originated from the recorded primary cancer. The flowchart of the study was shown in Figure 1.

### The Information of Primary Cancer and Non-cancer Cause of Death

The information on the primary cancer was derived from the variable of “Site recode ICD-O-3/WHO 2008” in the SEER^*^Stat software. The original site groups and the corresponding ICD-O-3 Site were acquired from the SEER website (https://seer.cancer.gov/siterecode/icdo3_dwhoheme/). Some types of cancers were integrated according to annual incidence. Other undefined primary cancers were classified into the “Miscellaneous” groups. The classification of primary cancer in the current study was shown in Supplementary Table 1.

The COD were classified according to the variable of “COD to site recode,” which was defined based on the International Statistical Classification of Diseases and Related Health Problems, Tenth Revision (ICD-10) codes. The deaths caused by tumors were attributed to cancer causes. Non-cancer causes were integrated and classified. The classification and corresponding codes of non-cancer causes in the current study were seen in Table 1.

**Table 1:** The classification of non-cancer causes in the present study.

**Classification**	**Cause of death definition**	**ICD-10 corresponding codes**
Cardiovascular and cerebrovascular	Diseases of heart	I00-I09, I11, I13, I20-I51
disease	Hypertension without heart disease	I10, I12
	Cerebrovascular diseases	I60-I69
	Atherosclerosis	I70
	Aortic aneurysm and dissection	I71
	Other diseases of arteries, arterioles, capillaries	I72-I78
Other causes	*In situ*, benign or unknown behavior neoplasm	D00-D48
	Complications of pregnancy, childbirth, puerperium	A34, O00-O95, O98-O99
	Congenital anomalies	Q00-Q99
	Certain conditions originating in perinatal period	P00-P96
	Symptoms, signs and ill-defined conditions	R00-R99
	Other cause of death	–
COPD and associated conditions	Chronic obstructive pulmonary disease and allied cond	J40-J47
Septicemia, infectious and	Tuberculosis	A15-A19
parasitic diseases	Syphilis	A50-A53
	Septicemia	A40-A41
	Other infectious and parasitic diseases including HIV	A00-A08, A20-A33, A35-A39, A42-A49, A54-B19, B25-B99
Pneumonia and influenza	Pneumonia and influenza	J09-J18
Accidents and adverse effects	Accidents and adverse effects	V01-X59, Y85-Y86
Diabetes	Diabetes mellitus	E10-E14
Nephritis, nephrotic syndrome	Nephritis, nephrotic syndrome and nephrosis	N00-N07, N17-N19, N25-N27
and nephrosis		
Suicide and self-inflicted injury	Suicide and self-inflicted injury	U03, X60-X84, Y87.0
Alzheimers	Alzheimers (ICD-9 and 10 only)	G30
Chronic liver disease and cirrhosis	Chronic liver disease and cirrhosis	K70, K73-K74
Stomach and duodenal ulcers	Stomach and duodenal ulcers	K25-K28
Homicide and legal intervention	Homicide and legal intervention	U01-U02, X85-Y09, Y35, Y87.1, Y89.0

### Statistics Analysis

The death latency was defined according to the reported survival of BM patients in previous studies (9, 12). In the present study, nearly 94% of non-cancer caused deaths occurred within 3 years after BM diagnosis. Thus, four intervals based on the latency from diagnosis to death were identified: <6 months, 6–11 months, 12–35 months, and >3 years. To adjust for gender, age and race, the stratified analysis was performed. Patients were divided into three groups according to age at initial diagnosis: 18–39 years, 40–59 years, and older than 59 years.

### Ethics Statement

We did not obtain the patients' consent because the SEER is an open database and the cancer is reportable disease allover the USA. This study is in accordance with the 1964 Declaration of Helsinki and subsequent amendments or comparable ethical standards.

## Results

### Baseline Characteristics of Patients With Non-cancer COD

**Figure 2:**
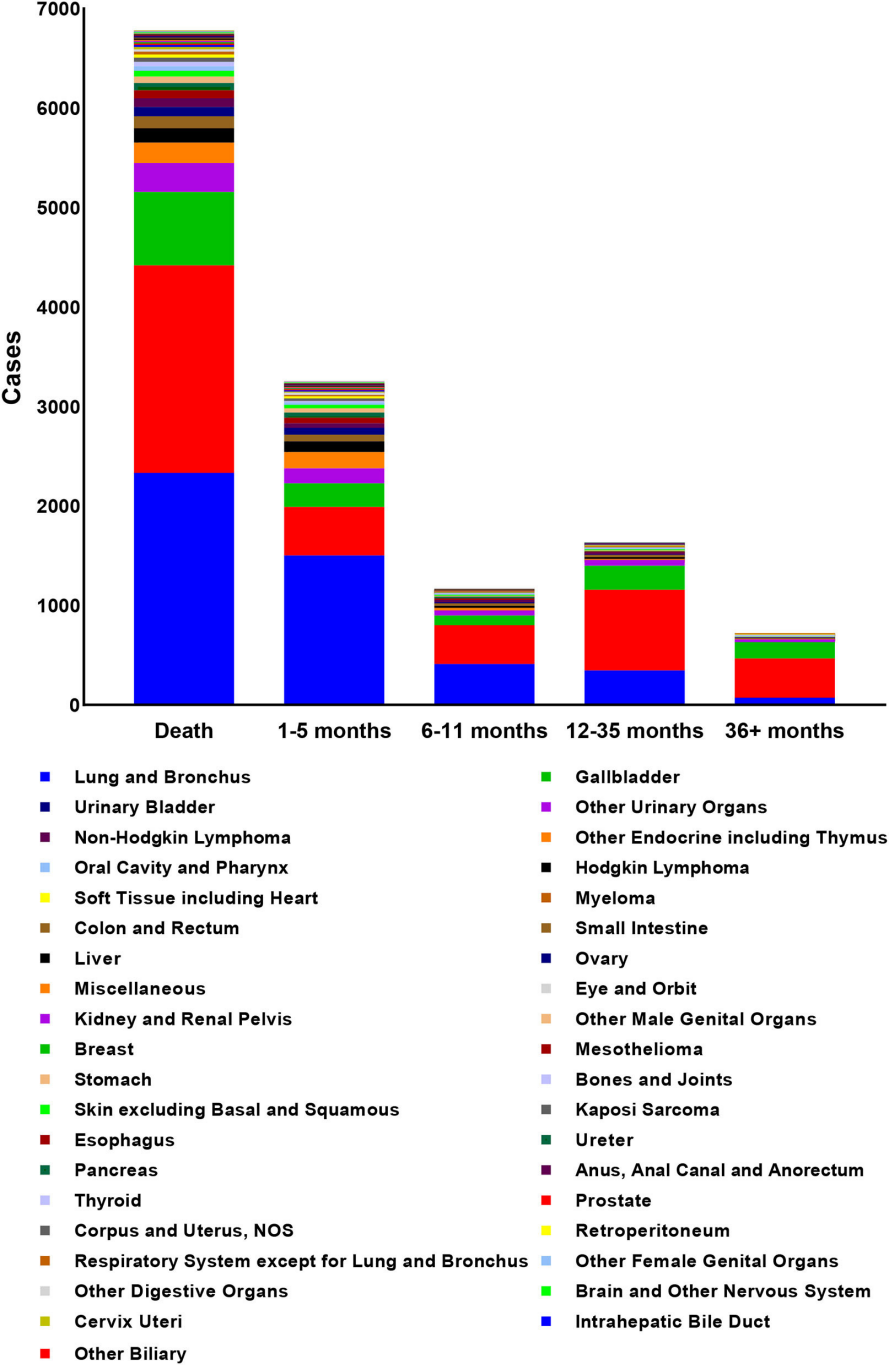
The deaths among different primary cancers and different death latencies.

According to the inclusion criteria, a total of 97,997 patients were included and 6,782 of them died of non-cancer causes. The mean age at death of non-cancer caused deaths was 75.58 years with male predominance (*N* = 4,515, 66.6%). The characteristics of patients with non-cancer COD and the number of deaths in different latency was shown in Table 2. The number of death cases in 18–39 years group, 40–59 years group and older than 59 years group were 76, 982, and 5,724, respectively. The primary cancer, lung and bronchus cancer, prostate cancer, breast cancer, kidney and renal pelvis cancer, and liver cancer all contributed top five deaths among all types of primary cancer. The deaths among different primary cancer were seen in Figure 2.

**Table 2:** Baseline characteristics of cancer patients with bone metastasis who died of non-cancer causes and of those who died according to the time of death after initial diagnosis.

**Characteristics**	**Death**	**Mean age at death**	**Deaths by time after BM diagnosis**			
			**1–5 months**	**6–11 months**	**12–35 months**	**36+ months**
**Total cohort**	6,782	72.58	3,254 (48.0%)	1,173 (17.3%)	1,634 (24.1%)	721 (10.6%)
**Age at diagnosis**						
18–39	76	33.35	40 (52.6%)	14 (18.4%)	14 (18.4%)	8 (10.5%)
40–59	982	54.21	517 (52.6%)	176 (17.9%)	213 (21.7%)	76 (7.7%)
≥60	5,724	76.25	2,697 (47.1%)	983 (17.2%)	1,407 (24.6%)	637 (11.1%)
**Gender**						
Male	4,515	73.41	2,079 (46.0%)	810 (17.9%)	1,146 (25.4%)	480 (10.6%)
Female	2,267	70.93	1,175 (51.8%)	363 (16%)	488 (21.5%)	241 (10.6%)
**Race**						
White	5,135	73.43	2,470 (48.1%)	884 (17.2%)	1,235 (24.1%)	546 (10.6%)
Black	1,180	68.88	572 (48.5%)	221 (18.7%)	278 (23.6%)	109 (9.2%)
Others	467	72.57	212 (45.4%)	68 (14.6%)	121 (25.9%)	66 (14.1%)
**Primary tumor**						
Lung and bronchus	2,332	70.29	1,502 (64.4%)	411 (17.6%)	347 (14.9%)	72 (3.1%)
Prostate	2,086	78.69	489 (23.4%)	390 (18.7%)	811 (38.9%)	396 (19%)
Breast	739	70.57	237 (32.1%)	98 (13.3%)	241 (32.6%)	163 (22.1%)
Kidney and renal pelvis	290	70.87	151 (52.1%)	51 (17.6%)	59 (20.3%)	29 (10.0%)
Miscellaneous	206	74.00	164 (79.6%)	26 (12.6%)	10 (4.9%)	6 (2.9%)
Liver	145	65.28	107 (73.8%)	20 (13.8%)	17 (11.7%)	1 (0.7%)
Colon and rectum	120	67.30	67 (55.8%)	25 (20.8%)	23 (19.2%)	5 (4.2%)
Urinary bladder	93	70.34	69 (74.2%)	12 (12.9%)	6 (6.5%)	6 (6.5%)
Non-hodgkin lymphoma	89	69.20	46 (51.7%)	23 (25.8%)	20 (22.5%)	0
Esophagus	80	67.53	56 (70.0%)	16 (20.0%)	8 (10.0%)	0
Pancreas	73	67.99	52 (71.2%)	16 (21.9%)	3 (4.1%)	2 (2.7%)
Stomach	64	65.74	42 (65.6%)	8 (12.5%)	10 (15.6%)	4 (6.2%)
Skin excluding basal and squamous	58	69.22	35 (60.3%)	11 (19.0%)	10 (17.2%)	2 (3.4%)
Oral cavity and pharynx	46	64.81	24 (52.2%)	9 (19.6%)	8 (17.4%)	5 (10.9%)
Thyroid	45	71.30	14 (31.1%)	9 (20.0%)	9 (20.0%)	13 (28.9%)
Corpus and uterus, NOS	42	71.09	27 (64.3%)	7 (16.7%)	5 (11.9%)	3 (7.1%)
Soft tissue including heart	31	68.27	22 (71.0%)	3 (9.7%)	3 (9.7%)	3 (9.7%)
Respiratory system except for lung and bronchus	28	62.57	12 (42.9%)	2 (7.1%)	9 (32.1%)	5 (17.9%)
Other digestive organs	25	70.82	23 (92.0%)	0	2 (8.0%)	0
Cervix uteri	22	60.79	12 (54.5%)	3 (13.6%)	7 (31.8%)	0
Intrahepatic bile duct	20	64.47	13 (65.0%)	3 (15.0%)	4 (20.0%)	0
Other biliary	16	63.96	11 (68.8%)	3 (18.8%)	2 (12.5%)	0
Gallbladder	13	68.61	7 (53.8%)	3 (23.1%)	3 (23.1%)	0
Other urinary organs	12	74.55	8 (66.7%)	1 (8.3%)	3 (25.0%)	0
Other endocrine including thymus	12	64.37	6 (50.0%)	2 (16.7%)	0	4 (33.3%)
Hodgkin lymphoma	12	60.77	7 (58.3%)	5 (41.7%)	0	0
Small intestine	11	62.23	7 (63.6%)	2 (18.2%)	2 (18.2%)	0
Ovary	11	81.93	7 (63.6%)	1 (9.1%)	2 (18.2%)	1 (9.1%)
Anus, anal canal and anorectum	10	60.95	6 (60.0%)	1 (10.0%)	3 (30.0%)	0
Mesothelioma	10	75.18	8 (80.0%)	1 (10%)	1 (10.0%)	0
Ureter	8	83.71	4 (50.0%)	2 (25.0%)	2 (25.0%)	0
Other male genital organs	7	54.09	3 (42.9%)	4 (57.1%)	0	0
Brain and other nervous system	6	74.04	5 (83.3%)	1 (16.7%)	0	0
Other female genital organs	6	73.38	4 (66.7%)	1 (16.7%)	1 (16.7%)	0
Bones and joints	5	56.56	4 (80.0%)	0	1 (20.0%)	0
Kaposi sarcoma	4	42.38	1 (25.0%)	2 (50.0%)	1 (25.0%)	0
Retroperitoneum	2	59.35	1 (50.0%)	0	0	1 (50.0%)
Myeloma	2	79.75	1 (50.0%)	1 (50.0%)	0	0
Eye and orbit	1	26.91	0	0	1 (100.0%)	0
Peritoneum, omentum and mesentery	0	–	0	0	0	0
Leukemia	0	–	0	0	0	0

### Non-cancer Causes Among Different Latency After BM Diagnosis

**Figure 3:**
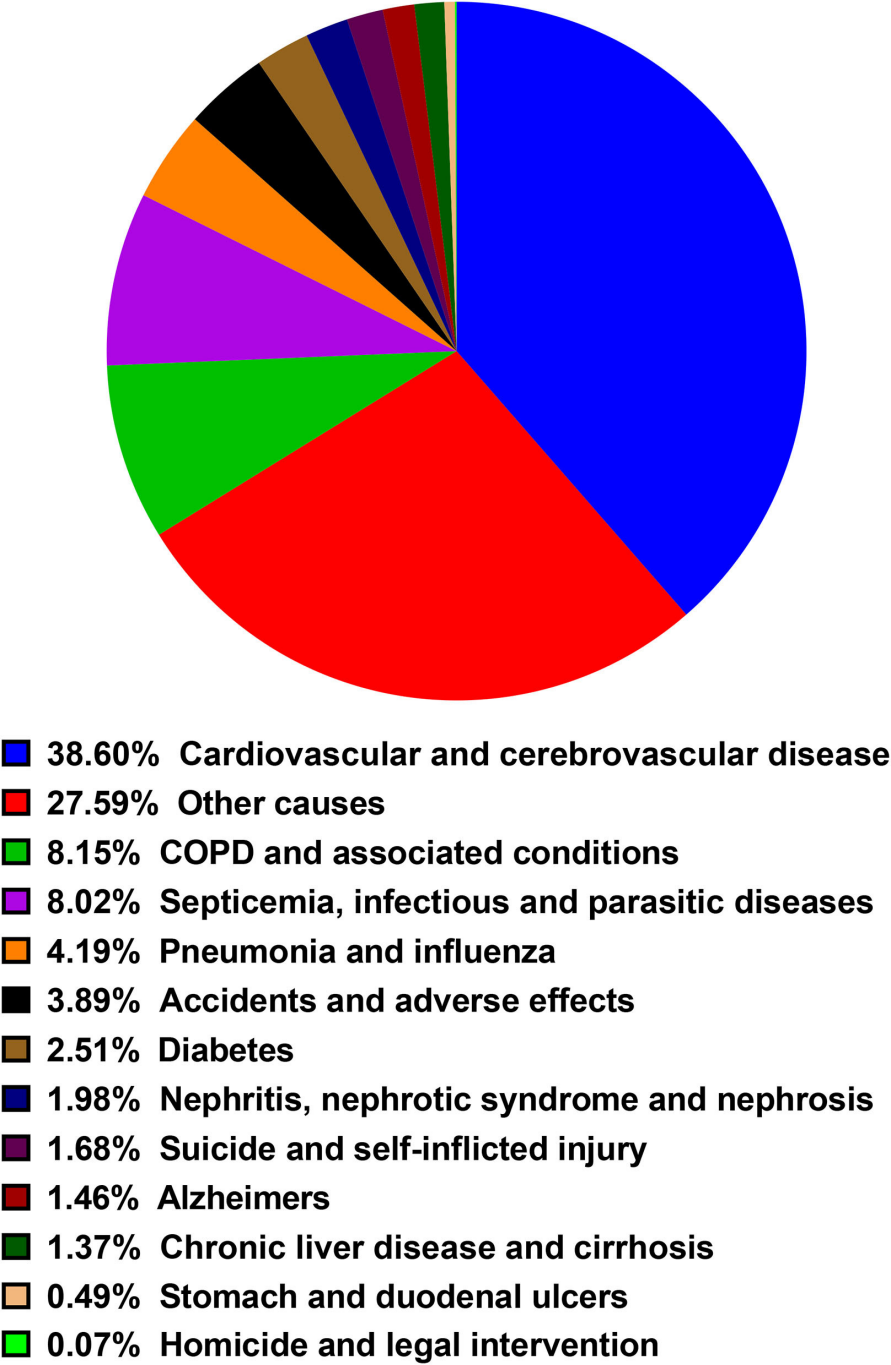
The proportion of each non-cancer causes of death (COD) in the present study.

**Figure 4:**
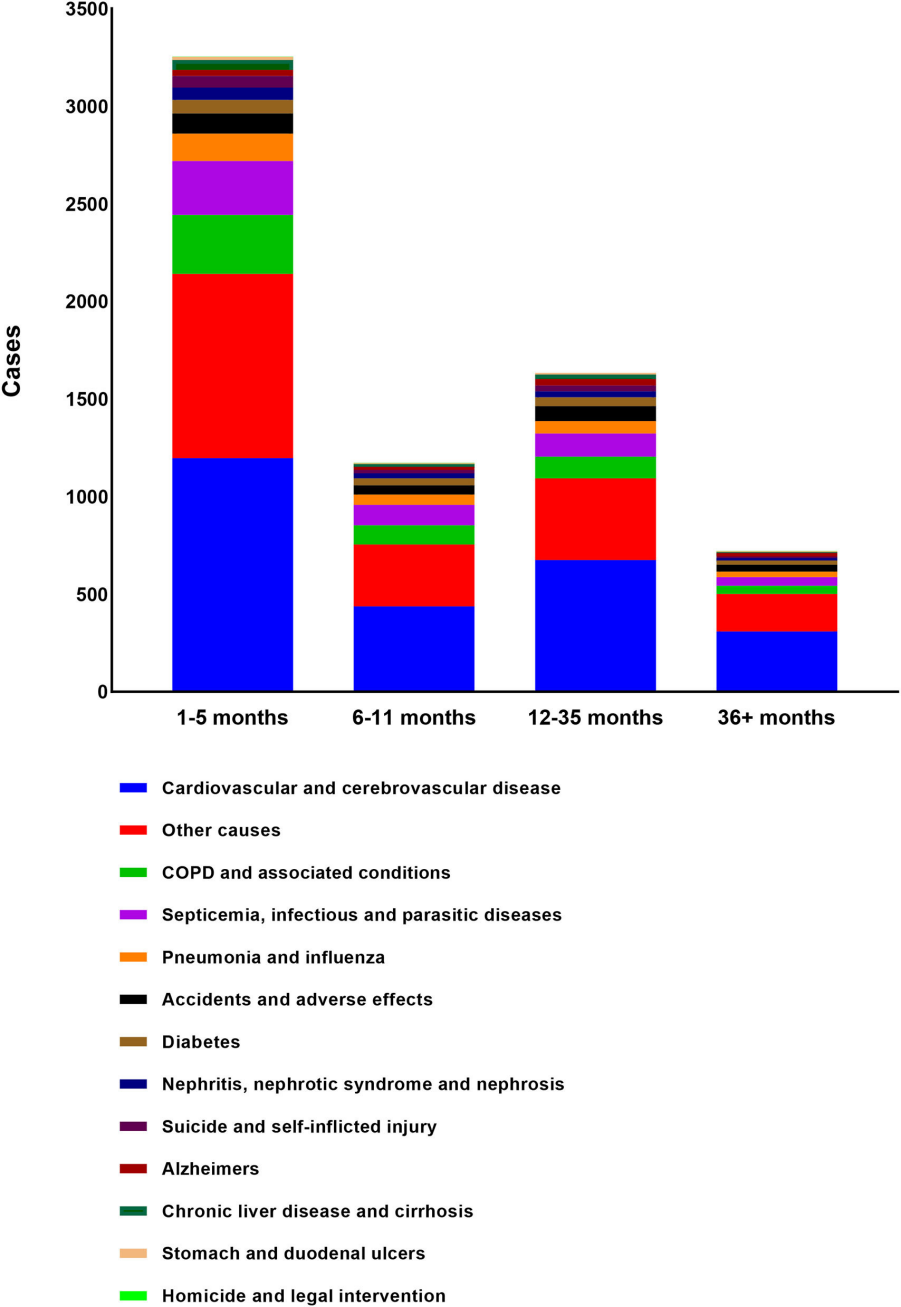
The frequencies of non-cancer COD among different death latency.

Almost half of deaths (*N* = 3,254, 48.0%) occurred within 6 months after BM diagnosis. A total of 2,807 deaths (41.4%) occurred from 6 months to 3 years after the BM diagnosis while 721 occurred more than 3 years after BM diagnosis (10.6%). The most common non-cancer cause of death was cardiovascular and cerebrovascular disease, followed by COPD and associated conditions, Septicemia, infectious and parasitic diseases, Pneumonia and influenza, Accidents, and adverse effects. The proportion of each non-cancer cause was shown in the diagram (Figure 3). As shown in Table 3, cardiovascular and cerebrovascular diseases were the leading non-cancer cause in all of latency. Among the patients who were dead within 6 months, COPD and associated conditions were the second non-cancer cause. Among the patients who lived longer than 6 months, septicemia, infectious and parasitic diseases was the second non-cancer cause. The frequencies of non-cancer causes among different death latency were given in Table 3 and Figure 4.

**Table 3:** Causes of death according to the time of death after initial BM diagnosis.

**Cause of death**	**Total death**	**Death by time after BM diagnosis**			
		**1–5 months**	**6–11 months**	**12–35 months**	**36+ months**
**All death**	97,997	50,257 (51.3%)	19,900 (20.3%)	21,955 (22.4%)	5,885 (6.0%)
**Cancer causes**	91,215	47,003 (51.5%)	18,727 (20.5%)	20,321 (22.3%)	5,164 (5.7%)
**Non-cancer causes**	6,782	3,254 (48.0%)	1,173 (17.3%)	1,634 (24.1%)	721 (10.6%)
Cardiovascular and cerebrovascular disease	2,618	1,197 (45.7%)	437 (16.7%)	675 (25.8%)	309 (11.8%)
Other causes	1,871	943 (50.4%)	318 (17.0%)	418 (22.3%)	192 (10.3%)
COPD and associated conditions	553	302 (54.6%)	98 (17.7%)	111 (20.1%)	42 (7.6%)
Septicemia, infectious and parasitic diseases	544	277 (50.9%)	104 (19.1%)	119 (21.9%)	44 (8.1%)
Pneumonia and influenza	284	140 (49.3%)	53 (18.7%)	63 (22.2%)	28 (9.9%)
Accidents and adverse effects	264	103 (39%)	48 (18.2%)	77 (29.2%)	36 (13.6%)
Diabetes	170	69 (40.6%)	35 (20.6%)	45 (26.5%)	21 (12.4%)
Nephritis, nephrotic syndrome and nephrosis	134	64 (47.8%)	26 (19.4%)	31 (23.1%)	13 (9.7%)
Suicide and self-inflicted injury	114	59 (51.8%)	17 (14.9%)	30 (26.3%)	8 (7.0%)
Alzheimers	99	32 (32.3%)	16 (16.2%)	33 (33.3%)	18 (18.2%)
Chronic liver disease and cirrhosis	93	50 (53.8%)	15 (16.1%)	23 (24.7%)	5 (5.4%)
Stomach and duodenal ulcers	33	16 (48.5%)	5 (15.2%)	8 (24.2%)	4 (12.1%)
Homicide and legal intervention	5	2 (40.0%)	1 (20.0%)	1 (20.0%)	1 (20.0%)

### Non-cancer COD in Different Gender

A total of 57,931 death occurred in male patients and 4,515 died of non-cancer causes. The most common non-cancer cause in men was cardiovascular and cerebrovascular diseases, followed by septicemia, infectious and parasitic diseases and COPD and associated conditions. Trends in CODs among different death latency were similar (Supplementary Table 2). The proportion of each non-cancer cause in male patients and the frequencies of non-cancer causes among different death latency were shown in Supplementary Figure 1.

A total of 40,066 death occurred in female patients, 2,267 of them died of non-cancer causes while others died of cancer. The most common non-cancer cause in women was cardiovascular and cerebrovascular diseases, followed by COPD and associated conditions and septicemia, infectious and parasitic disease. A similar trends in CODs among different death latencies were shown in Supplementary Table 3 and Supplementary Figure 2.

### Non-cancer COD in Different Age

As shown in Supplementary Table 4, a total of 1,527 cases died of cancer in the 18–39 age group and non-cancer causes contributed to 4.7% (*N* = 76). Septicemia and infectious and parasitic diseases were the most common non-cancer causes and almost all deaths (93.8%) occurred within 3 years after diagnosis. The secondary non-cancer cause was cardiovascular and cerebrovascular disease and all the death occurred within 3 years. The proportion of each non-cancer causes and the frequencies of non-cancer causes among different death latency were shown in Supplementary Figure 3.

There were 982 and 5,724 patients who died of non-cancer causes in 40–59 age group and older than 59 years group, respectively. Cardiovascular and cerebrovascular diseases were the leading non-cancer cause in patients aged 40–59 years. Septicemia and infectious and parasitic diseases were the second non-cancer cause. As for patients older than 59 years, COPD and associated conditions were the second non-cancer cause after cardiovascular and cerebrovascular diseases. More information among different death latency was shown in Supplementary Tables 5, 6 and Supplementary Figures 4, 5 (Supplementary Table 5; Supplementary Figure 4 for patients aged 40–59 years).

### Non-cancer COD in Different Race

As shown in Supplementary Table 7 and Supplementary Figure 6, a total of 5,135 cases died of non-cancer causes in white patients. Cardiovascular and cerebrovascular diseases were the most common non-cancer cause, which occurred in 1,943 patients. COPD and associated conditions were the second non-cancer cause in white patients, followed by septicemia, and infectious and parasitic diseases. The similar trend was shown in black patients. Cardiovascular and cerebrovascular diseases, septicemia, infectious and parasitic diseases, COPD, and associated conditions were the top three non-cancer causes for black patients (Supplementary Table 8; Supplementary Figure 7). As for American Indian/AK Native and Asian/Pacific Islander patients, pneumonia and influenza were the second non-cancer cause of death, which was different from other races. More information among different death latency was shown in Supplementary Table 9 and Supplementary Figure 8.

## Discussion

Our study showed that a total of 6,782 death of non-cancer causes and the mean age at death was 75.58 years old. Most deaths occurred within the first 3 years after diagnosis and in patients aged more than 60 years. The male represented a higher percentage than women in our cohort. Lung and bronchus cancer, prostate cancer, breast cancer, kidney and renal pelvis cancer, and liver cancer were responsible for the five major deaths among all types of primary cancers. Cardiovascular and cerebrovascular diseases were the most common non-cancer cause in both men and women, the 40–59 age group, and the older than 59 years group. To our knowledge, this is the first study to assess the non-cancer-related COD in patients with synchronous BM.

Cancer care has been facing several challenges in diagnosis, treatment, and follow-up all over the world. One of the most important challenges is the financial burden resulting from the advanced cancer care. The financial burden may significantly increase the mortality rate not only from the cancer-specific COD but also from the non-cancer causes (13–15). Thus, it is crucial to redirect the available resources toward all the COD in cancer patients with BM.

The management of BM may include surgery, radiation therapy, bone-targeted therapy with bisphosphonates or denosumab, and management of the known primary tumors with chemotherapy depending on the tumor types (16). However, the cardiovascular system is highly susceptible to the adverse effects of these treatments. It was reported that ~10–30% of patients who received radiation therapy developed the radiation-induced cardiac disease after 5–10 years of treatment (17). Chemotherapy can cause arrhythmias, angina or myocardial infarction, pericardial diseases, and cardiomyopathy with or without congestive heart failure (17). Our results postulated the upper hand of cardiovascular and cerebrovascular diseases as a non-cancer-related cause of death in patients with BM, which was consistent with previous studies. Batra et al. concluded that patients with lung cancer and pre-existing cardiovascular disease had a higher risk of dying from non-cancer causes than cancer causes (18). Compared to the general population, the SMR of fatal heart disease in cancer patients was up to 2.24 (95% *CI*: 2.23–2.25) (19). Older age, male, African American, and unmarried status were risk factors of fatal heart disease (19). A similar analysis was performed to identify cancer patients at high risk of fatal stroke, and the SMR of fatal stroke was 2.17 (95% *CI*: 2.15–2.19) compared to the general US population (20). An observational study based on the SEER database concluded that the mortality of cardiovascular disease was time-dependent in cancer patients and the first year after diagnosis was at high risk of dying from cardiovascular disease (21). Therefore, extensive discussion with the patients should be conducted before starting the therapy, and routine surveillance with cardiac imaging should be considered in long-term cancer survivors (22).

Other causes defined in our study included *in situ*, benign, or unknown behavior neoplasm; complications of pregnancy, childbirth, puerperium; congenital anomalies; certain conditions originating in the perinatal period; symptoms, signs, and ill-defined conditions; other COD. This category presented the second leading cause of non-cancer-related death and was observed mainly in patients aged <3 years. Besides, we observed that lung and bronchus cancer presented the vast majority of patients. The patients with primary tumors from respiratory diseases are most likely to have underlying COPD as both share a common risk factor in cigarette smoking; thus, COPD and associated conditions represented the third leading cause of non-cancer death in patients with BM. A total of 119,228 COPD deaths in cancer patients were recorded from 1975 to 2016 in the SEER database. Compared with the general population, cancer patients presented a close two-fold of risk at COPD deaths (23). Patients with lung cancer had a higher risk of COPD deaths than other extrapulmonary cancer patients (23). Chemotherapy and radiotherapy can also cause pulmonary toxicity, e.g., eosinophilic pneumonia, hypersensitivity pneumonitis, interstitial pneumonitis, non-cardiogenic pulmonary edema, and acute respiratory distress syndrome (24).

Septicemia, infectious and parasitic diseases was the third most common non-cancer-related death. Cancer patients suffer from immunosuppression due to the alteration of the phagocytic activity of the neutrophils and monocytes through chemo- and radiotherapy (25). Cytostatic drugs and monoclonal antibodies alter lymphocytes and natural killer cells and prolong lymphopenia (26, 27). This alteration in immunity in cancer patients made them liable to sepsis with associated high mortality (28). Zheng et al. reported the mortality rate of fatal infections in cancer patients was 260.1/100,000 person-years and the rate has been decreasing in recent years (29). Van de Louw et al. found 14.8, 30, and 46% hospital mortality with isolated sepsis, severe sepsis, and septic shock, respectively, in 119,379 cancer patients (30). Williams et al. found that there were 126,209 sepsis cases in cancer patients annually in the USA. The patients had a 37.8% in-hospital mortality and were much more likely to be hospitalized than non-cancer patients (31).

The disparities in cancer mortality and survival were identified among different races (32). The study concluded that non-Hispanic Whites had a better 5-year survival rate than other races (32). However, a retrospective study based on multiethnic cohort indicated the survival difference could be eliminated after adjusting by obesity (33). Comorbidities and treatment resistance associated with obesity could explain the survival difference in races (34). Except for obesity, socioeconomic factors, which include poverty, inadequate education, lack of health insurance, played an important role in prevention, early detection, and treatment of cancer. Thus, it was essential to analyze non-cancer COD in different race groups.

Our results were consistent with the current literature. Ye et al. found that cardiovascular causes followed by infectious diseases and respiratory diseases were the leading cause of deaths between 2006 and 2013 in their Australian population-based study. The risk of death from cardiovascular diseases was even higher in the first year of diagnosis (*SMR* = 1.44; 95% *CI*: 1.26–1.64) (35). Also, Afifi et al. reported that cardiovascular disease, cerebrovascular disease, and COPD were the most common non-cancer cause of death after 1 year from the diagnosis breast cancer (36). With the improvement of treatment strategies, cancer-specific survival has been increasing and there was a great decrease in cancer-caused death. Non-cancer deaths had become the major cause of death, especially in some types of cancer (37). Wang et al. reported that the non-cancer cause was the dominant cause of death (10,387 out of 22,386) in patients with thyroid cancer. The proportion of thyroid-caused death and other cancer-caused death were 31.0 and 22.6%, respectively (38). Based on 834,510 colorectal cancer patients, Chen et al. concluded that the mortality risk from non-cancer cause increased. Compared with the general US population, the SMR of non-cancer deaths was 2.02 (95% *CI*: 2.23–2.25) among colorectal cancer patients (39).

Our study is limited by the bias associated with the SEER database, including the retrospective nature of the analysis and lack of external validity of the result as the SEER database included patients in the USA only. Another limitation is that some COD may be underestimated or not reported; those causes were reported under “other causes” in the present study. They presented the second leading cause of non-cancer death; therefore, further research is needed to explore those causes.

In summary, lung and bronchus cancer was the most common cancer type resulting in the non-cancer caused death, followed by prostate cancer and breast cancer. Non-cancer causes in cancer patients with synchronous BM were represented the significant death causes, especially cardiovascular and cerebrovascular disease. Other non-cancer causes were found, such as COPD, septicemia, infectious, and parasitic diseases. The differences in frequencies and proportion were found between male patients and female patients. Such differences can be also found in different ages and races. These findings should be considered by physicians. Physicians can counsel cancer patients with BM regarding survivorship with death causes screening and focus on the prevention of non-cancer deaths.

## Data Availability Statement

Publicly available datasets were analyzed in this study. This data can be found here: The Surveillance, Epidemiology, and End Results (SEER) Program of the National Cancer Institute (NCI).

## Author Contributions

YX, WM, and CZ conceptualized and designed the study. BA and KA provided analyzed, interpreted data, and drafted the manuscript. YL and HW provided statistical support. FZ, KP, VC, SL, and HL reviewed the framework and content of the discussion. WM and CZ provided continuous supervision. All authors contributed to the article and approved the submitted version.

## Funding

The present study was sponsored by the Natural Science Foundation of China (82011530050, 81801781, and 82072004) and Laboratory of Tumor Immunology and Pathology (Army Medical University), Ministry of Education (2021jsz704).

## Conflict of Interest

The authors declare that the research was conducted in the absence of any commercial or financial relationships that could be construed as a potential conflict of interest.

## Publisher's Note

All claims expressed in this article are solely those of the authors and do not necessarily represent those of their affiliated organizations, or those of the publisher, the editors and the reviewers. Any product that may be evaluated in this article, or claim that may be made by its manufacturer, is not guaranteed or endorsed by the publisher.

## Supplementary Material

Supplementary Figure 1 The proportion of each non-cancer cause and the frequencies of non-cancer causes among different death latency in male patients.

Supplementary Figure 2 The proportion of each non-cancer cause and the frequencies of non-cancer causes among different death latency in female patients.

Supplementary Figure 3 The proportion of each non-cancer cause and the frequencies of non-cancer causes among different death latency in 18–39 age group.

Supplementary Figure 4 The proportion of each non-cancer cause and the frequencies of non-cancer causes among different death latency in 40–59 age group.

Supplementary Figure 5 The proportion of each non-cancer cause and the frequencies of non-cancer causes among different death latency in older than 59 age group.

Supplementary Figure 6 The proportion of each non-cancer cause and the frequencies of non-cancer causes among different death latency in White patients.

Supplementary Figure 7 The proportion of each non-cancer cause and the frequencies of non-cancer causes among different death latency in Black patients.

Supplementary Figure 8 The proportion of each non-cancer cause and the frequencies of non-cancer causes among different death latency in American Indian/AK Native and Asian/Pacific Islander patients.
