# Supplementary figures and images for: Non-cancer Causes of Death Following Initial Synchronous Bone Metastasis in Cancer Patients
Source: Front Med (Lausanne). 2022 Jun 2;9:899544. doi: 10.3389/fmed.2022.899544 (PMC9201113; doi:10.3389/fmed.2022.899544)

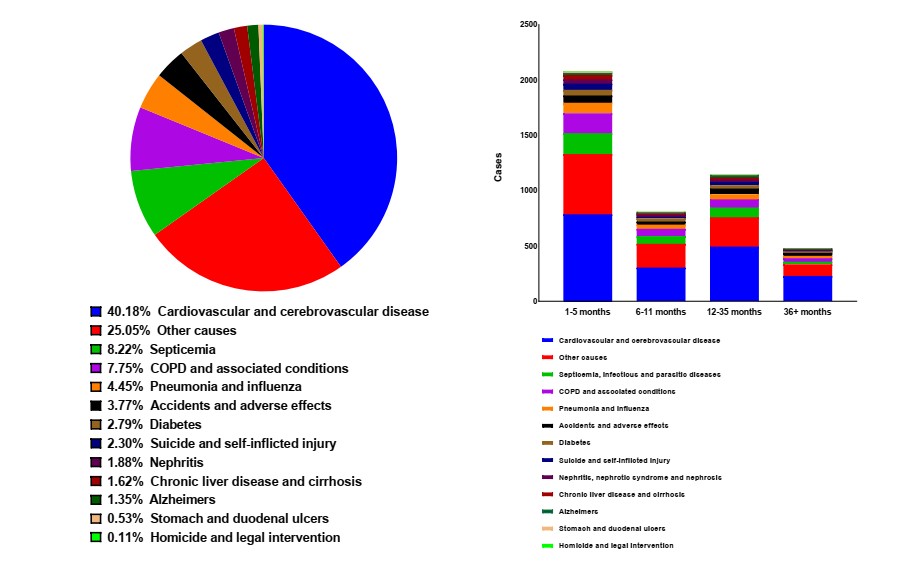

Supplement: Supplementary Figure 1 — The proportion of each non-cancer cause and the frequencies of non-cancer causes among different death latency in male patients. [file Image_1.JPEG]

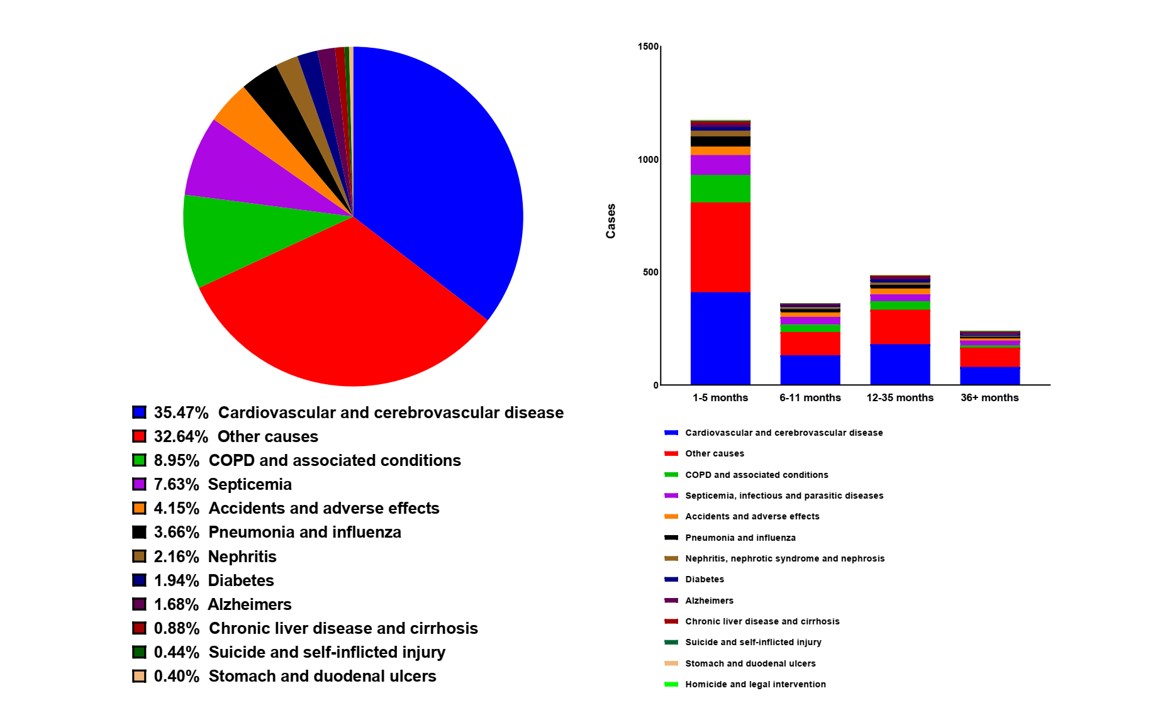

Supplement: Supplementary Figure 2 — The proportion of each non-cancer cause and the frequencies of non-cancer causes among different death latency in female patients. [file Image_2.JPEG]

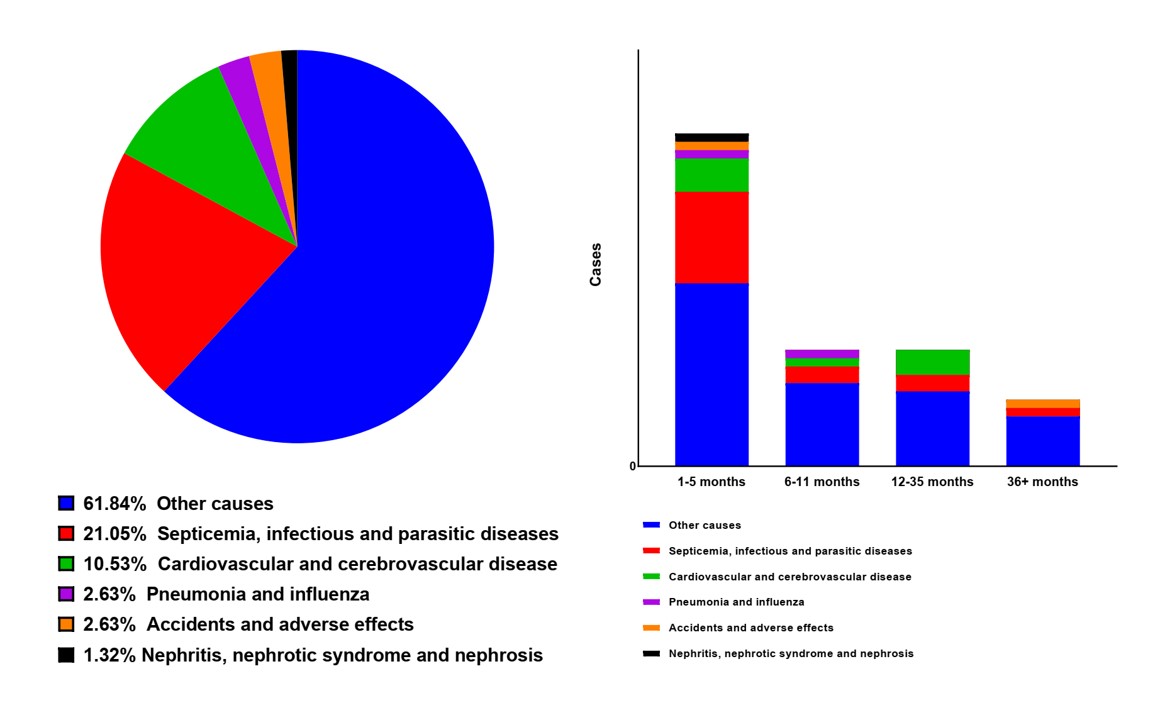

Supplement: Supplementary Figure 3 — The proportion of each non-cancer cause and the frequencies of non-cancer causes among different death latency in 18–39 age group. [file Image_3.JPEG]

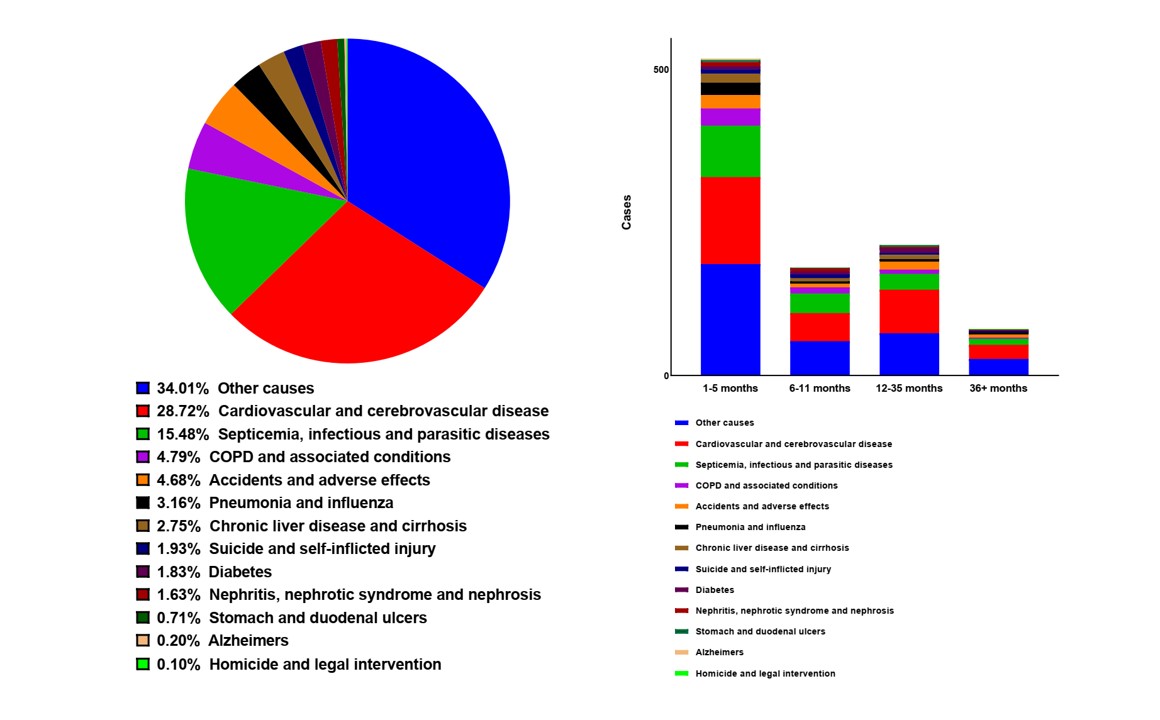

Supplement: Supplementary Figure 4 — The proportion of each non-cancer cause and the frequencies of non-cancer causes among different death latency in 40–59 age group. [file Image_4.JPEG]

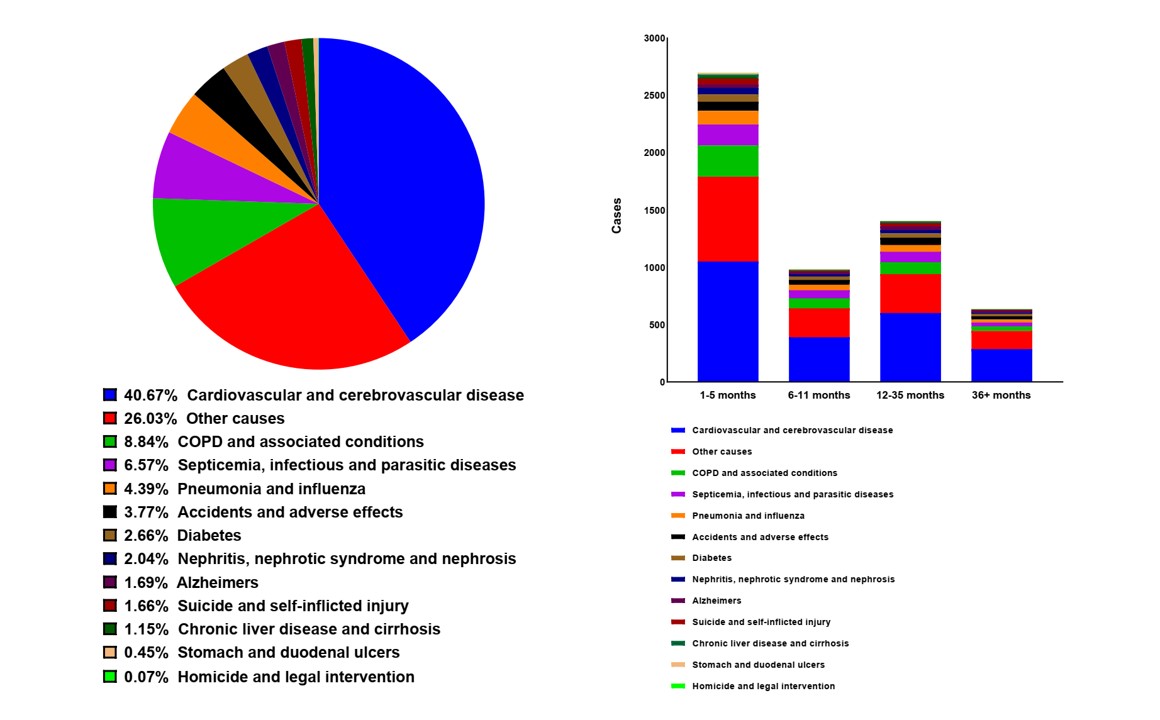

Supplement: Supplementary Figure 5 — The proportion of each non-cancer cause and the frequencies of non-cancer causes among different death latency in older than 59 age group. [file Image_5.JPEG]

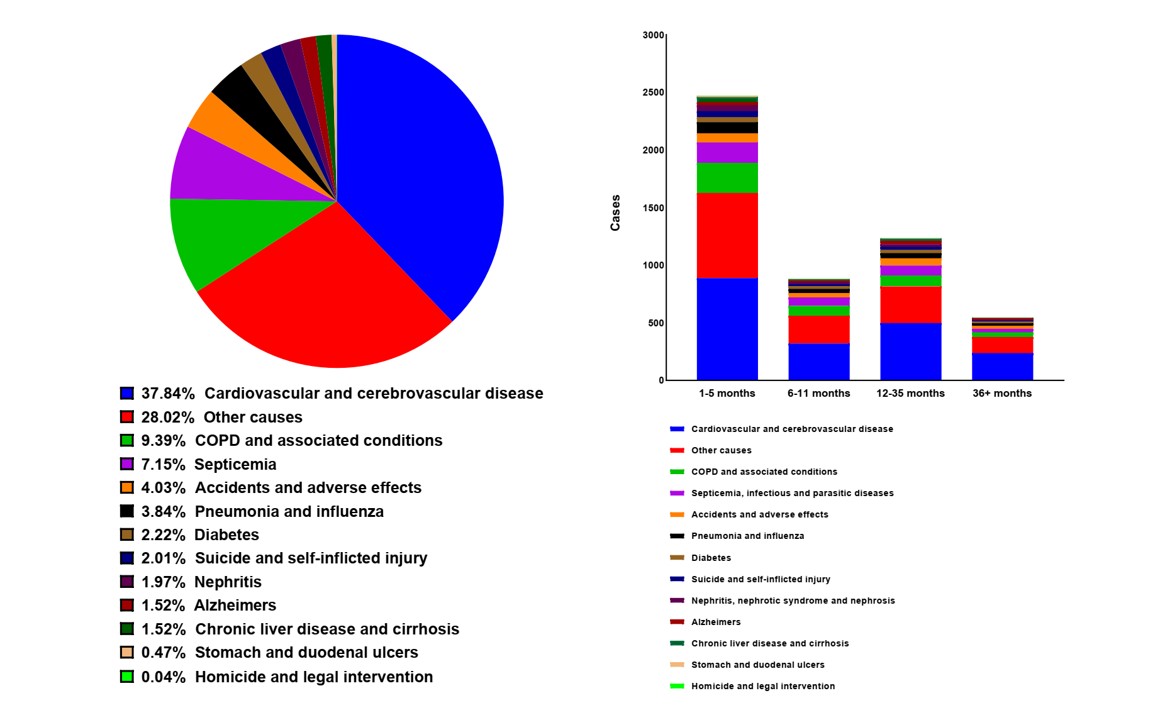

Supplement: Supplementary Figure 6 — The proportion of each non-cancer cause and the frequencies of non-cancer causes among different death latency in White patients. [file Image_6.JPEG]

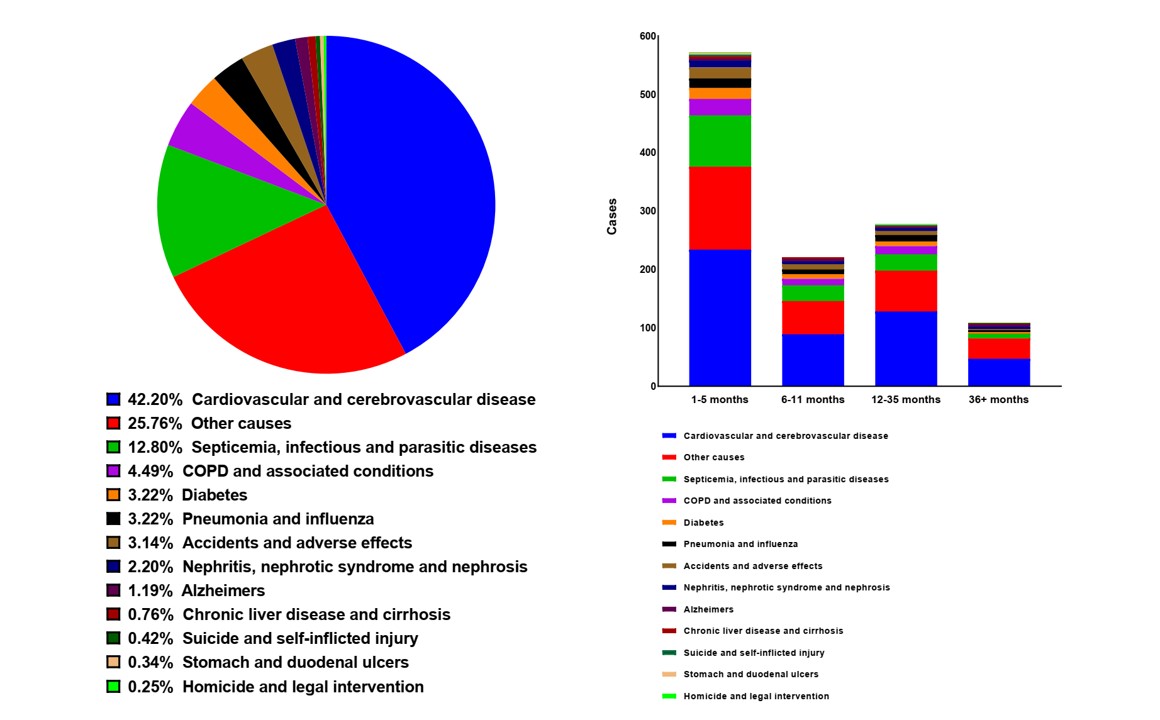

Supplement: Supplementary Figure 7 — The proportion of each non-cancer cause and the frequencies of non-cancer causes among different death latency in Black patients. [file Image_7.JPEG]

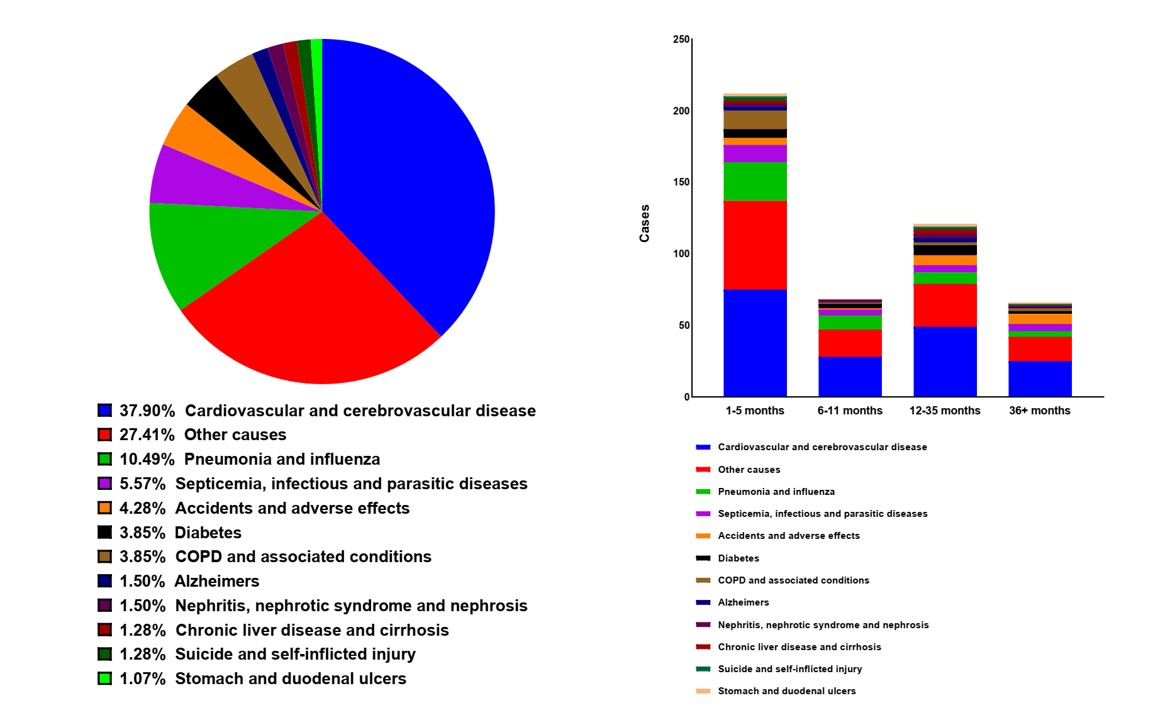

Supplement: Supplementary Figure 8 — The proportion of each non-cancer cause and the frequencies of non-cancer causes among different death latency in American Indian/AK Native and Asian/Pacific Islander patients. [file Image_8.JPEG]
