# Supplementary material for: Non-cancer Causes of Death Following Initial Synchronous Bone Metastasis in Cancer Patients
Source: Front Med (Lausanne). 2022 Jun 2;9:899544. doi: 10.3389/fmed.2022.899544 (PMC9201113; doi:10.3389/fmed.2022.899544)
Supplement: Supplementary file 9 [file Table_1.DOCX]

**Supplementary Table 1. The classification of primary cancer in the present study.**

| **Site Group in SEER database** | **Classification in the current study** | **Including** |
| --- | --- | --- |
| **Oral Cavity and Pharynx** | Oral Cavity and Pharynx | Lip, Tongue, Salivary Gland, Floor of Mouth, Gum and Other Mouth, Nasopharynx, Tonsil, Oropharynx, Hypopharynx, Other Oral Cavity and Pharynx |
| **Digestive System** | Esophagus |  |
|  | Stomach |  |
|  | Small Intestine |  |
|  | Colon and Rectum | Cecum, Appendix, Ascending Colon, Hepatic Flexure, Transverse Colon, Splenic Flexure, Descending Colon, Sigmoid Colon, Large Intestine, NOS, Rectosigmoid Junction, Rectum |
|  | Anus, Anal Canal and Anorectum |  |
|  | Liver |  |
|  | Intrahepatic Bile Duct |  |
|  | Gallbladder |  |
|  | Other Biliary |  |
|  | Pancreas |  |
|  | Retroperitoneum |  |
|  | Peritoneum, Omentum and Mesentery |  |
|  | Other Digestive Organs |  |
| **Respiratory System** | Lung and Bronchus |  |
|  | Respiratory System except for Lung and Bronchus | Nose, Nasal Cavity and Middle Ear; Larynx; Pleura; Trachea, Mediastinum and Other Respiratory Organs |
| **Bones and Joints** | Bones and Joints |  |
| **Soft Tissue including Heart** | Soft Tissue including Heart |  |
| **Skin excluding Basal and Squamous** | Skin excluding Basal and Squamous |  |
| **Breast** | Breast |  |
| **Female Genital System** | Cervix Uteri |  |
|  | Corpus and Uterus, NOS | Corpus Uteri; Uterus, NOS |
|  | Ovary |  |
|  | Other Female Genital Organs | Vagina; Vulva; Other Female Genital Organs |
| **Male Genital System** | Prostate |  |
|  | Other Male Genital Organs | Testis; Penis; Other Male Genital Organs |
| **Urinary System** | Urinary Bladder |  |
|  | Kidney and Renal Pelvis |  |
|  | Ureter |  |
|  | Other Urinary Organs |  |
| **Eye and Orbit** | Eye and Orbit |  |
| **Brain and Other Nervous System** | Brain and Other Nervous System |  |
| **Endocrine System** | Thyroid |  |
|  | Other Endocrine including Thymus |  |
| **Lymphoma** | Hodgkin Lymphoma |  |
|  | Non-Hodgkin Lymphoma |  |
| **Myeloma** | Myeloma |  |
| **Leukemia** | Leukemia |  |
| **Mesothelioma** | Mesothelioma |  |
| **Kaposi Sarcoma** | Kaposi Sarcoma |  |
| **Miscellaneous** | Miscellaneous |  |
