# Supplementary material for: Non-cancer Causes of Death Following Initial Synchronous Bone Metastasis in Cancer Patients
Source: Front Med (Lausanne). 2022 Jun 2;9:899544. doi: 10.3389/fmed.2022.899544 (PMC9201113; doi:10.3389/fmed.2022.899544)
Supplement: Supplementary file 10 [file Table_2.DOCX]

**Supplementary Table 2. Cancer causes and non-cancer causes of death according to the time of death after initial diagnosis in male patients.**

| **Cause of death** | **Total death** | **Death by time after BM diagnosis** | | | |
| --- | --- | --- | --- | --- | --- |
|  |  | **1-5 months** | **6-11 months** | **12-35 months** | **36+ months** |
| **All death** | 57931 | 29774 (51.4%) | 12014 (20.7%) | 12952 (22.4%) | 3191 (5.5%) |
| **Cancer causes** | 53416 | 27695 (51.8%) | 11204 (21%) | 11806 (22.1%) | 2711 (5.1%) |
| **Non-cancer causes** | 4515 | 2079 (46.0%) | 810 (17.9%) | 1146 (25.4%) | 480 (10.6%) |
| Cardiovascular and cerebrovascular disease | 1814 | 786 (43.3%) | 305 (16.8%) | 494 (27.2%) | 229 (12.6%) |
| Other causes | 1131 | 545 (48.2%) | 215 (19%) | 265 (23.4%) | 106 (9.4%) |
| Septicemia, infectious and parasitic diseases | 371 | 189 (50.9%) | 70 (18.9%) | 89 (24.0%) | 23 (6.2%) |
| COPD and associated conditions | 350 | 180 (51.4%) | 65 (18.6%) | 73 (20.9%) | 32 (9.1%) |
| Pneumonia and influenza | 201 | 95 (47.3%) | 38 (18.9%) | 47 (23.4%) | 21 (10.4%) |
| Accidents and adverse effects | 170 | 65 (38.2%) | 28 (16.5%) | 51 (30.0%) | 26 (15.3%) |
| Diabetes | 126 | 52 (41.3%) | 28 (22.2%) | 31 (24.6%) | 15 (11.9%) |
| Suicide and self-inflicted injury | 104 | 55 (52.9%) | 15 (14.4%) | 29 (27.9%) | 5 (4.8%) |
| Nephritis, nephrotic syndrome and nephrosis | 85 | 39 (45.9%) | 17 (20.0%) | 21 (24.7%) | 8 (9.4%) |
| Chronic liver disease and cirrhosis | 73 | 40 (54.8%) | 13 (17.8%) | 18 (24.7%) | 2 (2.7%) |
| Alzheimers | 61 | 18 (29.5%) | 11 (18.0%) | 22 (36.1%) | 10 (16.4%) |
| Stomach and duodenal ulcers | 24 | 13 (54.2%) | 4 (16.7%) | 5 (20.8%) | 2 (8.3%) |
| Homicide and legal intervention | 5 | 2 (40.0%) | 1 (20.0%) | 1 (20.0%) | 1 (20.0%) |
