# Supplementary material for: Non-cancer Causes of Death Following Initial Synchronous Bone Metastasis in Cancer Patients
Source: Front Med (Lausanne). 2022 Jun 2;9:899544. doi: 10.3389/fmed.2022.899544 (PMC9201113; doi:10.3389/fmed.2022.899544)
Supplement: Supplementary file 11 [file Table_3.DOCX]

**Supplementary Table 3. Cancer causes and non-cancer causes of death according to the time of death after initial diagnosis in female patients.**

| **Cause of death** | **Total death** | **Death by time after BM diagnosis** | | | |
| --- | --- | --- | --- | --- | --- |
|  |  | **1-5 months** | **6-11 months** | **12-35 months** | **36+ months** |
| **All death** | 40066 | 20483 (51.1%) | 7886 (19.7%) | 9003 (22.5%) | 2694 (6.7%) |
| **Cancer causes** | 37799 | 19308 (51.1%) | 7523 (19.9%) | 8515 (22.5%) | 2453 (6.5%) |
| **Non-cancer causes** | 2267 | 1175 (51.8%) | 363 (16.0%) | 488 (21.5%) | 241 (10.6%) |
| Cardiovascular and cerebrovascular disease | 804 | 411 (51.1%) | 132 (16.4%) | 181 (22.5%) | 80 (10.0%) |
| Other causes | 740 | 398 (53.8%) | 103 (13.9%) | 153 (20.7%) | 86 (11.6%) |
| COPD and associated conditions | 203 | 122 (60.1%) | 33 (16.3%) | 38 (18.7%) | 10 (4.9%) |
| Septicemia, infectious and parasitic diseases | 173 | 88 (50.9%) | 34 (19.7%) | 30 (17.3%) | 21 (12.1%) |
| Accidents and adverse effects | 94 | 38 (40.4%) | 20 (21.3%) | 26 (27.7%) | 10 (10.6%) |
| Pneumonia and influenza | 83 | 45 (54.2%) | 15 (18.1%) | 16 (19.3%) | 7 (8.4%) |
| Nephritis, nephrotic syndrome and nephrosis | 49 | 25 (51.0%) | 9 (18.4%) | 10 (20.4%) | 5 (10.2%) |
| Diabetes | 44 | 17 (38.6%) | 7 (15.9%) | 14 (31.8%) | 6 (13.6%) |
| Alzheimers | 38 | 14 (36.8%) | 5 (13.2%) | 11 (28.9%) | 8 (21.1%) |
| Chronic liver disease and cirrhosis | 20 | 10 (50.0%) | 2 (10.0%) | 5 (25.0%) | 3 (15.0%) |
| Suicide and self-inflicted injury | 10 | 4 (40.0%) | 2 (20.0%) | 1 (10.0%) | 3 (30.0%) |
| Stomach and duodenal ulcers | 9 | 3 (33.3%) | 1 (11.1%) | 3 (33.3%) | 2 (22.2%) |
| Homicide and legal intervention | 0 | 0 | 0 | 0 | 0 |
