# Supplementary material for: Non-cancer Causes of Death Following Initial Synchronous Bone Metastasis in Cancer Patients
Source: Front Med (Lausanne). 2022 Jun 2;9:899544. doi: 10.3389/fmed.2022.899544 (PMC9201113; doi:10.3389/fmed.2022.899544)
Supplement: Supplementary file 12 [file Table_4.DOCX]

**Supplementary Table 4. Cancer causes and non-cancer causes of death according to the time of death after initial diagnosis in patients aged 18-39 years.**

| **Cause of death** | **Total death** | **Death by time after BM diagnosis** | | | |
| --- | --- | --- | --- | --- | --- |
|  |  | **1-5 months** | **6-11 months** | **12-35 months** | **36+ months** |
| **All death** | 1603 | 530 (33.1%) | 446 (27.8%) | 504 (31.4%) | 123 (7.7%) |
| **Cancer causes** | 1527 | 490 (32.1%) | 432 (28.3%) | 490 (32.1%) | 115 (7.5%) |
| **Non-cancer causes** | 76 | 40 (52.6%) | 14 (18.4%) | 14 (18.4%) | 8 (10.5%) |
| Other causes | 47 | 22 (46.8%) | 10 (21.3%) | 9 (19.1%) | 6 (12.8%) |
| Septicemia, infectious and parasitic diseases | 16 | 11 (68.8%) | 2 (12.5%) | 2 (12.5%) | 1 (6.2%) |
| Cardiovascular and cerebrovascular disease | 8 | 4 (50.0%) | 1 (12.5%) | 3 (37.5%) | 0 |
| Pneumonia and influenza | 2 | 1 (50.0%) | 1 (50.0%) | 0 | 0 |
| Accidents and adverse effects | 2 | 1 (50.0%) | 0 | 0 | 1 (50.0%) |
| Nephritis, nephrotic syndrome and nephrosis | 1 | 1 (100%) | 0 | 0 | 0 |
