# Supplementary material for: Non-cancer Causes of Death Following Initial Synchronous Bone Metastasis in Cancer Patients
Source: Front Med (Lausanne). 2022 Jun 2;9:899544. doi: 10.3389/fmed.2022.899544 (PMC9201113; doi:10.3389/fmed.2022.899544)
Supplement: Supplementary file 13 [file Table_5.DOCX]

**Supplementary Table 5. Cancer causes and non-cancer causes of death according to the time of death after initial diagnosis in patients aged 40-59 years.**

| **Cause of death** | **Total death** | **Death by time after BM diagnosis** | | | |
| --- | --- | --- | --- | --- | --- |
|  |  | **1-5 months** | **6-11 months** | **12-35 months** | **36+ months** |
| **All death** | 22584 | 10683 (47.3%) | 5152 (22.8%) | 5457 (24.2%) | 1292 (5.7%) |
| **Cancer causes** | 21602 | 10166 (47.1%) | 4976 (23.0%) | 5244 (24.3%) | 1216 (5.6%) |
| **Non-cancer causes** | 982 | 517 (52.6%) | 176 (17.9%) | 213 (21.7%) | 76 (7.7%) |
| Other causes | 334 | 182 (54.5%) | 56 (16.8%) | 69 (20.7%) | 27 (8.1%) |
| Cardiovascular and cerebrovascular disease | 282 | 142 (50.4%) | 46 (16.3%) | 71 (25.2%) | 23 (8.2%) |
| Septicemia, infectious and parasitic diseases | 152 | 84 (55.3%) | 32 (21.1%) | 26 (17.1%) | 10 (6.6%) |
| COPD and associated conditions | 47 | 28 (59.6%) | 10 (21.3%) | 7 (14.9%) | 2 (4.3%) |
| Accidents and adverse effects | 46 | 22 (47.8%) | 6 (13.0%) | 13 (28.3%) | 5 (10.9%) |
| Pneumonia and influenza | 31 | 20 (64.5%) | 4 (12.9%) | 4 (12.9%) | 3 (9.7%) |
| Chronic liver disease and cirrhosis | 27 | 15 (55.6%) | 5 (18.5%) | 7 (25.9%) | 0 (0%) |
| Suicide and self-inflicted injury | 19 | 7 (36.8%) | 6 (31.6%) | 3 (15.8%) | 3 (15.8%) |
| Diabetes | 18 | 5 (27.8%) | 5 (27.8%) | 8 (44.4%) | 0 |
| Nephritis, nephrotic syndrome and nephrosis | 16 | 7 (43.8%) | 5 (31.2%) | 2 (12.5%) | 2 (12.5%) |
| Stomach and duodenal ulcers | 7 | 3 (42.9%) | 1 (14.3%) | 3 (42.9%) | 0 (0%) |
| Alzheimers | 2 | 2 (100%) | 0 | 0 | 0 |
| Homicide and legal intervention | 1 | 0 | 0 | 0 | 1 (100%) |
