# Supplementary material for: Non-cancer Causes of Death Following Initial Synchronous Bone Metastasis in Cancer Patients
Source: Front Med (Lausanne). 2022 Jun 2;9:899544. doi: 10.3389/fmed.2022.899544 (PMC9201113; doi:10.3389/fmed.2022.899544)
Supplement: Supplementary file 14 [file Table_6.DOCX]

**Supplementary Table 6. Cancer causes and non-cancer causes of death according to the time of death after initial diagnosis in patients older than 60 years.**

| **Cause of death** | **Total death** | **Death by time after BM diagnosis** | | | |
| --- | --- | --- | --- | --- | --- |
|  |  | **1-5 months** | **6-11 months** | **12-35 months** | **36+ months** |
| **All death** | 73810 | 39044 (52.9%) | 14302 (19.4%) | 15994 (21.7%) | 4470 (6.1%) |
| **Cancer causes** | 68086 | 36347 (53.4%) | 13319 (19.6%) | 14587 (21.4%) | 3833 (5.6%) |
| **Non-cancer causes** | 5724 | 2697 (47.1%) | 983 (17.2%) | 1407 (24.6%) | 637 (11.1%) |
| Cardiovascular and cerebrovascular disease | 2328 | 1051 (45.1%) | 390 (16.8%) | 601 (25.8%) | 286 (12.3%) |
| Other causes | 1490 | 739 (49.6%) | 252 (16.9%) | 340 (22.8%) | 159 (10.7%) |
| COPD and associated conditions | 506 | 274 (54.2%) | 88 (17.4%) | 104 (20.6%) | 40 (7.9%) |
| Septicemia, infectious and parasitic diseases | 376 | 182 (48.4%) | 70 (18.6%) | 91 (24.2%) | 33 (8.8%) |
| Pneumonia and influenza | 251 | 119 (47.4%) | 48 (19.1%) | 59 (23.5%) | 25 (10.0%) |
| Accidents and adverse effects | 216 | 80 (37.0%) | 42 (19.4%) | 64 (29.6%) | 30 (13.9%) |
| Diabetes | 152 | 64 (42.1%) | 30 (19.7%) | 37 (24.3%) | 21 (13.8%) |
| Nephritis, nephrotic syndrome and nephrosis | 117 | 56 (47.9%) | 21 (17.9%) | 29 (24.8%) | 11 (9.4%) |
| Alzheimers | 97 | 30 (30.9%) | 16 (16.5%) | 33 (34.0%) | 18 (18.6%) |
| Suicide and self-inflicted injury | 95 | 52 (54.7%) | 11 (11.6%) | 27 (28.4%) | 5 (5.3%) |
| Chronic liver disease and cirrhosis | 66 | 35 (53.0%) | 10 (15.2%) | 16 (24.2%) | 5 (7.6%) |
| Stomach and duodenal ulcers | 26 | 13 (50.0%) | 4 (15.4%) | 5 (19.2%) | 4 (15.4%) |
| Homicide and legal intervention | 4 | 2 (50.0%) | 1 (25.0%) | 1 (25.0%) | 0 |
