# Supplementary material for: Non-cancer Causes of Death Following Initial Synchronous Bone Metastasis in Cancer Patients
Source: Front Med (Lausanne). 2022 Jun 2;9:899544. doi: 10.3389/fmed.2022.899544 (PMC9201113; doi:10.3389/fmed.2022.899544)
Supplement: Supplementary file 15 [file Table_7.DOCX]

**Supplementary Table 7. Cancer causes and non-cancer causes of death according to the time of death after initial diagnosis in White patients.**

| **Cause of death** | **Total death** | **Death by time after BM diagnosis** | | | |
| --- | --- | --- | --- | --- | --- |
|  |  | **1-5 months** | **6-11 months** | **12-35 months** | **36+ months** |
| **All death** | 77217 | 40128 (52%) | 15813 (20.5%) | 16810 (21.8%) | 4466 (5.8%) |
| **Cancer causes** | 72082 | 37658 (52.2%) | 14929 (20.7%) | 15575 (21.6%) | 3920 (5.4%) |
| **Non-cancer causes** | 5135 | 2470 (48.1%) | 884 (17.2%) | 1235 (24.1%) | 546 (10.6%) |
| Cardiovascular and cerebrovascular disease | 1943 | 888 (45.7%) | 320 (16.5%) | 498 (25.6%) | 237 (12.2%) |
| Other causes | 1439 | 739 (51.4%) | 242 (16.8%) | 318 (22.1%) | 140 (9.7%) |
| COPD and associated conditions | 482 | 261 (54.1%) | 86 (17.8%) | 95 (19.7%) | 40 (8.3%) |
| Septicemia, infectious and parasitic diseases | 367 | 177 (48.2%) | 73 (19.9%) | 86 (23.4%) | 31 (8.4%) |
| Accidents and adverse effects | 207 | 79 (38.2%) | 38 (18.4%) | 63 (30.4%) | 27 (13%) |
| Pneumonia and influenza | 197 | 97 (49.2%) | 35 (17.8%) | 44 (22.3%) | 21 (10.7%) |
| Diabetes | 114 | 44 (38.6%) | 24 (21.1%) | 30 (26.3%) | 16 (14%) |
| Suicide and self-inflicted injury | 103 | 53 (51.5%) | 17 (16.5%) | 26 (25.2%) | 7 (6.8%) |
| Nephritis, nephrotic syndrome and nephrosis | 101 | 50 (49.5%) | 19 (18.8%) | 23 (22.8%) | 9 (8.9%) |
| Alzheimers | 78 | 26 (33.3%) | 12 (15.4%) | 28 (35.9%) | 12 (15.4%) |
| Chronic liver disease and cirrhosis | 78 | 44 (56.4%) | 12 (15.4%) | 19 (24.4%) | 3 (3.8%) |
| Stomach and duodenal ulcers | 24 | 11 (45.8%) | 5 (20.8%) | 5 (20.8%) | 3 (12.5%) |
| Homicide and legal intervention | 2 | 1 (50.0%) | 1 (50.0%) | 0 | 0 |
