# Supplementary material for: Non-cancer Causes of Death Following Initial Synchronous Bone Metastasis in Cancer Patients
Source: Front Med (Lausanne). 2022 Jun 2;9:899544. doi: 10.3389/fmed.2022.899544 (PMC9201113; doi:10.3389/fmed.2022.899544)
Supplement: Supplementary file 16 [file Table_8.DOCX]

**Supplementary Table 8. Cancer causes and non-cancer causes of death according to the time of death after initial diagnosis in Black patients.**

| **Cause of death** | **Total death** | **Death by time after BM diagnosis** | | | |
| --- | --- | --- | --- | --- | --- |
|  |  | **1-5 months** | **6-11 months** | **12-35 months** | **36+ months** |
| **All death** | 13173 | 6559 (49.8%) | 2649 (20.1%) | 3093 (23.5%) | 872 (6.6%) |
| **Cancer causes** | 11993 | 5987 (49.9%) | 2428 (20.2%) | 2815 (23.5%) | 763 (6.4%) |
| **Non-cancer causes** | 1180 | 572 (48.5%) | 221 (18.7%) | 278 (23.6%) | 109 (9.2%) |
| Cardiovascular and cerebrovascular disease | 498 | 234 (47.0%) | 89 (17.9%) | 128 (25.7%) | 47 (9.4%) |
| Other causes | 304 | 142 (46.7%) | 57 (18.8%) | 70 (23.0%) | 35 (11.5%) |
| Septicemia, infectious and parasitic diseases | 151 | 88 (58.3%) | 27 (17.9%) | 28 (18.5%) | 8 (5.3%) |
| COPD and associated conditions | 53 | 28 (52.8%) | 11 (20.8%) | 14 (26.4%) | 0 |
| Diabetes | 38 | 19 (50.0%) | 8 (21.1%) | 8 (21.1%) | 3 (7.9%) |
| Pneumonia and influenza | 38 | 16 (42.1%) | 8 (21.1%) | 11 (28.9%) | 3 (7.9%) |
| Accidents and adverse effects | 37 | 19 (51.4%) | 9 (24.3%) | 7 (18.9%) | 2 (5.4%) |
| Nephritis, nephrotic syndrome and nephrosis | 26 | 12 (46.2%) | 5 (19.2%) | 5 (19.2%) | 4 (15.4%) |
| Alzheimers | 14 | 3 (21.4%) | 4 (28.6%) | 2 (14.3%) | 5 (35.7%) |
| Chronic liver disease and cirrhosis | 9 | 4 (44.4%) | 3 (33.3%) | 1 (11.1%) | 1 (11.1%) |
| Suicide and self-inflicted injury | 5 | 3 (60.0%) | 0 | 2 (40%) | 0 |
| Stomach and duodenal ulcers | 4 | 3 (75.0%) | 0 | 1 (25.0%) | 0 |
| Homicide and legal intervention | 3 | 1 (33.3%) | 0 | 1 (33.3%) | 1 (33.3%) |
