# Supplementary material for: Non-cancer Causes of Death Following Initial Synchronous Bone Metastasis in Cancer Patients
Source: Front Med (Lausanne). 2022 Jun 2;9:899544. doi: 10.3389/fmed.2022.899544 (PMC9201113; doi:10.3389/fmed.2022.899544)
Supplement: Supplementary file 17 [file Table_9.DOCX]

**Supplementary Table 9. Cancer causes and non-cancer causes of death according to the time of death after initial diagnosis in American Indian/AK Native and Asian/Pacific Islander patients.**

| **Cause of death** | **Total death** | **Death by time after BM diagnosis** | | | |
| --- | --- | --- | --- | --- | --- |
|  |  | **1-5 months** | **6-11 months** | **12-35 months** | **36+ months** |
| **All death** | 7607 | 3570 (46.9%) | 1438 (18.9%) | 2052 (27.0%) | 547 (7.2%) |
| **Cancer causes** | 7140 | 3358 (47.0%) | 1370 (19.2%) | 1931 (27.0%) | 481 (6.7%) |
| **Non-cancer causes** | 467 | 212 (45.4%) | 68 (14.6%) | 121 (25.9%) | 66 (14.1%) |
| Cardiovascular and cerebrovascular disease | 177 | 75 (42.4%) | 28 (15.8%) | 49 (27.7%) | 25 (14.1%) |
| Other causes | 128 | 62 (48.4%) | 19 (14.8%) | 30 (23.4%) | 17 (13.3%) |
| Pneumonia and influenza | 49 | 27 (55.1%) | 10 (20.4%) | 8 (16.3%) | 4 (8.2%) |
| Septicemia, infectious and parasitic diseases | 26 | 12 (46.2%) | 4 (15.4%) | 5 (19.2%) | 5 (19.2%) |
| Accidents and adverse effects | 20 | 5 (25.0%) | 1 (5.0%) | 7 (35.0%) | 7 (35.0%) |
| Diabetes | 18 | 6 (33.3%) | 3 (16.7%) | 7 (38.9%) | 2 (11.1%) |
| COPD and associated conditions | 18 | 13 (72.2%) | 1 (5.6%) | 2 (11.1%) | 2 (11.1%) |
| Alzheimers | 7 | 3 (42.9%) | 0 | 3 (42.9%) | 1 (14.3%) |
| Nephritis, nephrotic syndrome and nephrosis | 7 | 2 (28.6%) | 2 (28.6%) | 3 (42.9%) | 0 |
| Chronic liver disease and cirrhosis | 6 | 2 (33.3%) | 0 | 3 (50.0%) | 1 (16.7%) |
| Suicide and self-inflicted injury | 6 | 3 (50.0%) | 0 | 2 (33.3%) | 1 (16.7%) |
| Stomach and duodenal ulcers | 5 | 2 (40.0%) | 0 | 2 (40.0%) | 1 (20.0%) |
